# Profiting on Crisis: How Predatory Financial Investors Have Worsened Inequality in the Coronavirus Crisis

**DOI:** 10.1177/00027642211003162

**Published:** 2021-11

**Authors:** Megan Tobias Neely, Donna Carmichael

**Affiliations:** 1 Copenhagen Business School, Frederiksberg, Denmark; 2 London School of Economics, London, UK

**Keywords:** inequality, financialization, neoliberalism, crisis, COVID-19, financial services, shadow banking

## Abstract

A once-in-a-century pandemic has sparked an unprecedented health and economic crisis. Less examined is how predatory financial investors have shaped the crisis and profited from it. We examine how U.S. shadow banks, such as private equity, venture capital, and hedge fund firms, have affected hardship and inequality during the crisis. First, we identify how these investors helped to hollow out the health care industry and disenfranchise the low-wage service sector, putting frontline workers at risk. We then outline how, as the downturn unfolds, shadow banks are shifting their investments in ways that profit on the misfortunes of frontline workers, vulnerable populations, and distressed industries. After the pandemic subsides and governments withdraw stimulus support, employment will likely remain insecure, many renters will face evictions, and entire economic sectors will need to rebuild. Shadow banks are planning accordingly to profit from the fallout of the crisis. We argue that this case reveals how financial investors accumulate capital through private and speculative investments that exploit vulnerabilities in the economic system during a time of crisis. To conclude, we consider the prospects for change and inequality over time.

Since the late 1970s, the world financial system has become the dominant engine of the global economy (Dore, 2008; Epstein, 2005). The rise of finance has exacerbated inequality both within and between countries (Assa, 2012; Kus, 2013; Kwon & Roberts, 2015). This trend appears more pronounced in liberal market economies such as the United States (Kwon & Roberts, 2015), where finance has become a primary driver of rising top incomes and widening inequality (Lin & Neely, 2020; Lin & Tomaskovic-Devey, 2013).

The coronavirus pandemic presents an opportunity to change the unequal status quo. By creating a jolt to social and economic systems, moments of instability open up possibilities for change. As Raewyn Connell theorizes (2019), crises can expose cracks in the systems of inequality that allow people to create alternatives. However, recent economic crises have reinforced the uneven distribution of resources that favors finance, especially following the global financial crisis of 2008 (Grusky et al., 2011; Lin & Neely, 2020).

At first glance, the coronavirus crisis appears straightforward: A once-in-a-century pandemic sparked a health and economic crisis. But behind the scenes is a story of how financial investors have affected the crisis and profited from it. How has finance made society vulnerable during times of crisis? And how do financial investors exploit these vulnerabilities?

We examine how the rise of “shadow banks”—less regulated private credit intermediaries such as private equity, venture capital, and hedge fund firms—has shaped the course of hardship and inequality during the crisis. We focus on the United States because it has the highest concentration of shadow banks (Cox, 2019). Private equity invests in private companies and often proactively influences how executives run the company. Venture capital, a type of private equity, invests in start-up companies and provides guidance to entrepreneurs. Hedge funds invest in both public stock markets and private companies. These shadow banks play an instrumental role in how executives manage companies, which has important ramifications for societal responses to crises, the wellbeing and livelihoods of workers, and inequality throughout the labor market. Shadow banking is a highly lucrative sector of financial services responsible for driving top incomes ever further upward (Flaherty, 2015; Nau, 2013).

Shadow banking during the coronavirus crisis presents a case of capital accumulation through exploiting economic disruption. This case provides another way of conceptualizing David Harvey’s (2003) theory of global capital accumulation through dispossession, which occurs through financial wars against foreign currencies, productive economies, and corporations. In the coronavirus case, capital accumulation occurs through financial investors exploiting the economic system itself during a time of crisis. The coronavirus crisis is indicative of disaster capitalism (Klein, 2007) in which investors capitalize on a society characterized by unprecedented and unmanageable risk (Beck, 1992). Leading up to and during the coronavirus crisis, dispossession has involved the provision of public goods such as health care, health insurance, ambulatory services, and groceries. Financial investors at shadow banks have both created the conditions that exacerbated hardship during this crisis and invested in ways that have allowed them to profit from it. We call this profiting on crisis.

The stakes during the pandemic are high as people face a once-in-a-lifetime health crisis and economic disaster. To date, there have been over 119 million cases and 2.6 million deaths recorded worldwide, and the United States alone accounts for over 29.4 million cases and 530,000 deaths (“Coronavirus World Map,” 2021). The economic ramifications have been harrowing for communities and households. The World Bank reported a 4.3% contraction in global GDP at the end of 2020 (The World Bank, 2021). Employment remains insecure, many renters face evictions, and entire economic sectors struggle. Despite these conditions, U.S. financial markets continue to grow. This boom in market activity captures how a small group of investors is profiting from the crisis, while the majority of people face unprecedented health and economic hardships. This gap is indicative of widening inequality over the past four decades.

## The Rise of Finance and Widening Inequality

Since the late 1970s, deregulation of the U.S. financial sector has allowed finance to expand in leaps and bounds—and become increasingly risky, opaque, and complex (G. Davis, 2009; Krippner, 2005; Philippon & Reshef, 2013). Finance’s share of corporate profits tripled over the past 60 years, averaging 15% in the postwar era then peaking at 45% before the 2008 financial crisis (Krippner, 2005; Tomaskovic-Devey & Lin, 2011). During this same period, however, finance’s share of employment only increased from about 4% in 1950 to just over 7% in 2001 (Krippner, 2005). Thus, a smaller share of workers reaps the rewards of growth in the financial sector than did in the manufacturing sector’s heyday. The ability of financial actors to influence politics to favor deregulation, leverage bargaining power, and stimulate market demand has transferred large amounts of income to this sector (Hacker & Pierson, 2010; Lin & Neely, 2020; Tomaskovic-Devey & Lin, 2011).

As finance expanded its influence to other sectors of the economy, it brought about a widespread shift in corporate governance, called the shareholder value revolution. Before 1980, corporate management focused on reinvesting earnings to develop the company’s workers and products. After 1980, the dominant model of corporate governance understood the corporation’s primary purpose as promoting the interest of its shareholders, including executives, by maximizing profits (Fligstein & Shin, 2007; Ho, 2009; Lazonick & O’Sullivan, 2000). To maximize profits, financial investors have pressured companies to downsize, de-unionize, outsource, and computerize jobs (Jung, 2015). Rather than cutting costs, this focus on shareholder profit served to redistribute earnings from workers to managers, executives, and investors (Fligstein & Shin, 2007; Goldstein, 2012; Shin, 2014). Overall, this financial restructuring of the corporation resulted in less negotiating power, fewer protections, and greater insecurity for workers (Kalleberg, 2011; Lin & Neely, 2020).

The expanding size and power of finance has increased the concentration of capital into the hands of a select few and driven widening economic inequality (Lin & Neely, 2020; Philippon & Reshef, 2013). Three primary financial forces have exacerbated inequality (Lin & Neely, 2020). First, the shareholder value revolution has allowed finance to extract resources from the economy and weakened labor’s bargaining power relative to executives and financial investors. Second, financial professionals in the financial and nonfinancial sectors have devised new investment products that yield enormous profits (Godechot, 2016; Lin & Tomaskovic-Devey, 2013; Tomaskovic-Devey & Lin, 2011). These products have further weakened the demand for and power of other workers (Lin & Tomaskovic-Devey, 2013). Last, disparities in household lending allow the middle and upper class to leverage debt with low interest rates, while the working class, especially those who are families of color, struggle to obtain credit with higher interest rates and fees (Fligstein & Goldstein, 2015; Lin & Neely, 2020; Seamster & Charron-Chénier, 2017). Average households and workers struggle to make ends meet, while CEOs and Wall Street reap escalating top incomes, setting the stage for how each group has fared during the coronavirus pandemic.

## Instability and Crisis in the Neoliberal Era

Neoliberal policy regimes have been a primary driver in relaxing financial regulations and the subsequent rise of financial capitalism. Neoliberalism refers to a set of economic policies and practices guided by a market ideology that believes that markets have an internal stabilizing logic and less regulated, competitive markets will reach equilibrium (Harvey, 2007). Although neoliberal market activity is embedded within a particular regulatory and policy context (Brenner & Theodore, 2002; Sassen, 2004), financial markets can nimbly traverse a network’s “flow architecture” (Cetina, 2004) that can become divorced in speed and content from the underlying real economy. The rapid pace of financial markets promotes imitation and causes extreme price movements (MacKenzie, 2003), which heighten instability and lead to more frequent crises (Harvey, 2011).

The flexibility of global financial markets allows market actors to exploit the immobility of nations, firms, and workers (Boltanski & Chiapello, 2007). Investors can extract economic rents (Appelbaum, 2017; Tomaskovic-Devey & Lin, 2011; Weeden & Grusky, 2014)—returns on assets that owners control to ensure fixed supplies (Sorenson, 2000). Previously, capitalists created value by extracting physical capital and exploiting labor; now, value is also created by capitalizing on financial markets, such as through investments in foreign markets and derivatives vehicles (Hoang, 2018; Mackenzie, 2006). Foreign investors extract capital by dispossessing economies of raw materials and development opportunities (Harvey, 2003; Sassen, 2014); for example, providing loans with high fees and interest rates to foreign countries and betting against foreign currencies, causing them to collapse. We argue that the inherent instability of financial capitalism and its unprecedented risks (Beck, 1992; Harvey, 2011; Klein, 2007) provide new opportunities for investors to direct these techniques inward, capitalizing on vulnerabilities in their national economies rather than abroad.

Yet instability can create opportunities for systemic change. The decades following the Great Depression is called the “Great Compression” because of declining inequality (Goldin & Margo, 1992; Grusky et al., 2011). To a large extent, the policy response led to this compression: The New Deal introduced the federal minimum wage, higher taxation, stimulus spending, Social Security, and other welfare programs. In contrast, the policy response to the Great Recession boosted liquidity, penalized fraud, and reduced systemic risk, restoring the financial order that exacerbates inequality (Lin & Neely, 2020). To date, the government response to the pandemic has more closely followed the latter crisis with respect to the implications for inequality.

Policy, however, is only one piece of this puzzle. Thomas Piketty (2014) also identifies the bankruptcies of the Great Depression and destruction of physical capital during the World Wars as radical shocks that stemmed how inequality grows under rentier capitalism, in which income accrues disproportionately to those with capital. Physical capital has not been threatened on the same level during the coronavirus pandemic. But, we argue, the key difference between the shocks of past crises and the pandemic lies in how the current era of financial capitalism relies on exploiting labor and capital itself, through complex financial vehicles, for example, derivatives, that become divorced from the real economy. The activity of shadow banks during the pandemic reveals how financial investors accumulate capital through private and risky investments that exploit vulnerabilities in the economic system during a time of crisis. In a context of less regulatory scrutiny than in the postwar era, the opaque and speculative nature of shadow banking prevents the crisis from acting as a shock to capital-driven inequality, allowing inequality to grow rather than contract.

## The Case of Shadow Banking

Shadow banks have played an important role in the shareholder value revolution (Appelbaum & Batt, 2014) and contributed to the increasing polarization and precariousness of work (Kalleberg, 2011). Shadow banking refers to any kind of nondepository (i.e., nonbank) credit intermediation provided by asset managers (e.g., private equity, hedge fund, and venture capital firms), broker-dealers, and finance companies (Antill et al., 2014; International Monetary Fund, 2014; Pozsar et al., 2010). Shadow banks provide an opportune case for studying how finance has affected the current crisis for three primary reasons.

First, despite their enormous volume of assets and large impact on global capital flows, shadow banks make up some of the more complex, opaque, and less-regulated subsectors of finance (Antill et al., 2014). The nature of their investments makes shadow banks less transparent than investment banks. These firms provide credit through a wide range of complex financial vehicles called alternative assets, such as asset-backed securities, collateralized debt obligations, asset-backed commercial paper, and repurchase agreements. Furthermore, shadow banks invest in public markets, in private markets—direct investments in companies that have not gone public—or in a combination of both. Investments in private markets draw less scrutiny than those in public markets, making shadow banking activities less transparent.

Second, while shadow banking is relatively unknown among the public, it has been the fastest growing areas of finance since the early 1980s (Antill et al., 2014). Institutional investors and wealthy elites have reduced their traditional investments in public markets and increased their investments in shadow banks such as private equity, where returns have historically been higher (Mauboussin & Callahan, 2020).^1^ This trend intensified in the aftermath of the 2008 financial crisis. As investment banks faced liquidity problems, the U.S. government tightened banking regulations and the Federal Reserve lowered interest rates, leading institutional investors to shift their funds to shadow banks less encumbered by regulators (International Monetary Fund, 2014). Both in the previous and current crises, these firms have presented an alternative to the large investment banks and provided opportunities to make higher investment returns. Thus, the shadow banking industry grew substantially—by 75% to $52 trillion (Cox, 2019)—as investors lost trust in the “too big to fail” financial institutions in the aftermath of 2008. Shadow banking’s rapid growth paved the way for the current crisis.

Last, the nature of their investments and the potential to generate high profits make shadow banks ideal for studying how the financial sector contributes to inequality in the labor force and instability in the economy. Shadow banks often make rapid, short-term trades that can create bubbles and crashes, as in the mortgage crisis precipitating 2008 (Gorton & Metrick, 2009). Furthermore, leveraged buyout firms, distressed debt investors, and activist hedge funds profit from investments in flailing companies with the goal to turn them around, which can promote these companies’ growth but can make them more prone to bankruptcy and failure (Appelbaum & Batt, 2014). Shadow banks also invest with the goal of maximizing shareholder value, which weakens workers’ bargaining power, funnels money to executives and shareholders, and thus contributes to widening inequality (Appelbaum & Batt, 2014). Finally, shadow banks provide high earnings both to their employees and to their wealthy investors, as discussed above, increasing the top incomes and wealth concentration among elites. Shadow banks have contributed to widening inequality and increasingly precarious work over the past 40 years (Kalleberg, 2011; Lin & Neely, 2020).

## Findings

Behind the headlines of personal protective equipment (PPE) shortages and vaccine developments is the role of shadow banking in making society more vulnerable to crisis and then profiting from these very vulnerabilities. The work of shadow banks has impacted the health care sector’s ability to respond to the pandemic and exacerbated the burden on frontline workers. In what follows, we first examine how shadow banks in the United States place pressure on executives to make business decisions that can endanger the welfare of many people and social institutions designed to protect the collective wellbeing. These financial investors have worked to dismantle and privatize the institutions responsible for protecting people, both when they are in the labor force as well as when they have exited it, such as during periods of unemployment and sickness. In particular, shadow banking has contributed to efforts to hollow out the health care industry and disenfranchise the low-wage service sector, putting frontline workers at risk and inhibiting the societal response to the coronavirus crisis. The opacity and complexity of shadow banking suggest that this sector has the potential to do more damage than the traditional banking sector.

We then outline how, both before and during the coronavirus crisis, high-net-worth and institutional investors, such as endowments and pension funds, have increased and plan to continue increasing allocations to these industries. Pensions and company-sponsored retirement accounts do not cover everyone, leaving out many workers, especially those whose benefits have been cut due to the shareholder value revolution.

Last, we investigate how, as the downturn unfolds, investors are shifting their financial holdings to profit on the misfortunes of frontline workers, vulnerable populations, and distressed industries. For example, investors are speculating on mounting insurance premiums (Brush, 2020); flailing travel, leisure, retail, and restaurant industries (Chung, 2020); distressed real estate assets, such as retail and hospitality; and niche industrial spaces, such as self-storage, data centers, and medical offices (Gujral et al., 2020; Jacobius, 2021). After governments withdraw financial support, unemployment is likely to remain high and foreclosures and business bankruptcies will likely ensue. Investors are planning accordingly to profit on the crisis and the fallout of the crisis.

### Private Equity and Inequality

The expansion of financial services has increased the income and bargaining power of executives and investors at the middle and working class’s expense (G. Davis, 2009; Lin & Neely, 2020). At the forefront of this transformation has been private equity, which often reduces workers’ wages and benefits to extract economic value from the businesses they own (Appelbaum & Batt, 2014; Souleles, 2019). To improve the profitability of acquired companies, private equity owners change the management and business practices in ways that often have negative impacts on workers. Techniques include closing plants and facilities, laying off workers, automating jobs, offshoring and outsourcing labor, terminating collective bargaining agreements, shifting labor to nonunion facilities, and reducing employees’ wages and benefits, especially for unionized workers (Appelbaum & Batt, 2014). Following a private equity acquisition, workers who perform routine or offshorable work double their unemployment incidence, with even greater increases for those with aggressive labor unions (Olsson & Tåg, 2016). Private equity owners are even more likely to shut down plants and take other cost-reduction strategies during financial crises (S. Davis et al., 2014).

Those workers deemed “frontline” during the crisis—health care, grocery, and distribution workers—have been among those hardest hit by private equity in the years leading up to the crisis. Private equity has focused its efforts on the health care industry with drastic impacts on hospitals, urgent care, and ambulances (Appelbaum & Batt, 2014). Many private equity firms aim for a quick turnover in their acquisitions, making them less likely to invest in new technology, workers’ skills, quality improvements, and emergency equipment stockpiles (Appelbaum, 2019). This omission left hospital workers at risk when the coronavirus hit and the stocks of PPE were insufficient.

Furthermore, private equity has driven mergers and acquisitions that have increased hospital monopolies. This consolidation of hospitals has led to increased health care costs (Cooper et al., 2019) and overburdened yet underpaid health care staff (Garcia-Gomez et al., 2020; Kinard & Wright, 2011), making it harder to fill shifts when health care workers catch the highly contagious virus. These trends have led to closures of less profitable hospitals and other health care facilities, cutting off low-income and rural areas from access to health care. As the coronavirus crisis swept rural areas, hospitals became overwhelmed and at risk for bankruptcy without the steady income of elective surgeries covered by insurance (Boatright & Liedtke, 2020).

Private equity’s cost-cutting strategies have had adverse outcomes for first responders, too. Since the financial crisis of 2008, private equity has taken over ambulatory and fire-fighting services resulting in longer wait times, less reliable medical equipment, and poorer care overall (Ivory et al., 2016). These adverse outcomes have led some public officials to deem private equity a threat to public well-being and safety.

The private equity takeover of health care has put patients at risk of higher bills and medical debt. Patients are less likely to control which physicians treat them at the emergency room, increasing the likelihood that they will be charged high out-of-network and surprise bills (Appelbaum & Batt, 2014; Cooper et al., 2020). Eileen Applebaum and Rosemary Batt (2020) found that bills from the health care groups Envision Healthcare and TeamHealth, owned by the private equity behemoths Blackstone and K.K.R., accounted for considerable increases in surprise medical bills. Private equity buyouts also have had negative impacts on patient health and compliance with care standards at nursing homes (Gupta et al., 2020).

Health care workers are not the only frontline workers whom private equity has disadvantaged. Grocery store and other workers in the low-wage service sector are more likely to work part-time (and forced to juggle multiple jobs), have fewer benefits, and face unpredictable on-demand scheduling (Crain & Sherraden, 2014; Lambert, 2008). The 2.8 million grocery store chain workers are the most likely to be unionized among retail workers (Batt, 2018). Yet their livelihoods and stability have been compromised as 50 major grocery chains have been taken over by private equity, which has pushed executives to cut labor costs and shut down less profitable stores. Seven chains, that employ over 125,000 workers, have since filed for bankruptcy over the past 5 years. When bankruptcies arise, workers, vendors, suppliers, and landlords are the hardest hit in loss of jobs, income, and benefits. The coronavirus crisis occurred at a moment when grocery store jobs have become scarcer and less secure, making it difficult for workers to mobilize for protection from the health risks of working during the pandemic.

The health care and low-wage service sector workers that have been adversely affected by private equity are more likely to be women, especially women of color. One-third of jobs held by women are deemed “essential” (Robertson & Gebeloff, 2020). Women comprise 52% of frontline workers, including 9 out of 10 nurses and nursing assistants and two-thirds of checkout workers at grocery stores and pharmacies. The PPE shortfalls and underequipped hospitals and elder-care facilities have put these women at a higher risk of catching COVID-19 (Nguyen et al., 2020). Over 2,900 health care workers have died from COVID-19, and nurses—a job disproportionately held by women—are among those most vulnerable (Jewett et al., 2020). Meanwhile, women are more likely to be unemployed than men, and Latinx women have the highest rates of unemployment followed by Black women (Gezici & Ozay, 2020). The dire consequences of the private equity takeover during the pandemic have been uneven according to race, immigration, gender, and social class, because of inequality in the labor market and the commoditization of health care.

### Capital Flows into Shadow Banking

Against a backdrop of surging infection and mortality rates in the United States, stock markets are soaring as capital flows into public and private financial markets in anticipation of massive returns on investments in specific sectors that stand to benefit from the pandemic (Barro, 2020). Public markets, which hedge funds invest in, provide a barometer of these capital flows. The S&P 500, for example, took a dive in March and then rallied for months, closing 2020 at a record high with an annual increase of 16.3% despite the increasingly dire situation facing the health care sector and the accompanying economic fallout (Troise, 2020). Precipitous upticks in unemployment, business closures, and economic chaos would seem to be a recipe for dismal performance in stock prices; however, with historically low yields in cash and bond markets, investors are seeking investment opportunities in areas of finance where returns will skyrocket due to the pandemic. These wealthy individuals and institutional investors are flocking to shadow banking, in particular, because it is proactively sourcing opportunities emerging from the pandemic (Bernstein et al., 2019).

Indeed, investors’ appetite for shadow banking stands to grow considerably, with many expecting to increase their financial commitments, in spite (or perhaps because) of the uncertainties. Investors are motivated by the potentially higher financial returns offered by shadow banks, which can outperform investments in public stock markets, especially when interest rates are low. The assets managed by the alternative investment sector, a subset of shadow banks that includes hedge funds and private equity, ballooned to more than $10 trillion as yield-hungry investors poured capital into the industry during 2019 (Preqin, 2020a). These trends will likely continue as the pandemic plays its course. In a June 2020 survey, 63% of surveyed investors said the pandemic has not affected their planned alternatives commitments, with 29% looking to increase investments in alternatives (Preqin, 2020b).

The fact that shadow banks are emerging as a “winner” of the pandemic is not unique to this crisis. During the financial crisis of 2008, shadow banks were among the first to rebound and then profit from the crisis because they did not face the same liquidity problems as investment banks (Bernstein et al., 2019). Private equity firms and other shadow banks actually increased their fundraising during the last financial crisis as investors pulled their money from large financial institutions (Arundale & Mason, 2020; International Monetary Fund, 2014). It does not appear that the current crisis will hamper investment allocations to shadow banks in the long term.

Investors’ increasing allocations to shadow banking reflect broader trends over the past 30 years, as the industry has been the fastest growing in the financial sector (Antill et al., 2014). For example, from 1990 to 2007—just before the financial crisis—the assets managed globally by hedge funds grew 50-fold and have since grown by another third (“Hedge Funds in Trouble,” 2008). And these trends have continued in recent years. Globally, private equity fundraising reached $535 billion in 2020, with North America setting an all-time record of $350 billion in 2019 (McKinsey & Company, 2020a; Mendoza, 2021). Similarly, the value of venture capital deals skyrocketed to a 1-year record high of $156 billion in 2020 (*Venture Monitor*, 2020). “Dry powder,” large pools of capital set aside for rapidly emerging opportunities, is an important metric for shadow banks. In anticipation of pandemic-induced opportunities, investors are pouring capital into private investment vehicles, with total private capital dry powder reaching a record $2.4 trillion at mid-2020, a hundred billion dollar increase compared with the end of 2019. And, as we examine in the next section, hedge fund investments have been so profitable during the crisis that the industry is still growing despite investors redeeming up to $100 billion in 2020 (Laurelli, 2020).

The long-term growth of shadow banking, before and during the crisis, reflects how the sector has lobbied regulators and governments to change policy in their favor. For example, the National Venture Capital Association launched an initiative to promote the positive impact of venture capital on the coronavirus battle. The Association led a letter-writing campaign to the congressional leadership to communicate the “critical work that VC-backed start-ups are undertaking to advance cures, treatments, and products needed to fight COVID-19” (Temkin, 2020). Meanwhile, 8,100 venture and private equity portfolio companies received an estimated $13.4 billion in loans from the U.S. Paycheck Protection Program designed to incentivize small businesses to continue paying their employees (Thorne, 2020). Critics questioned whether companies with access to private funding and that constitute high-risk ventures should receive the government stimulus.

Last, the influx of capital to shadow banks during the pandemic has been funneled into the hands of an elite group of mostly white men, who not only make higher incomes from managing these assets but also make decisions that affect the rest of society. The firms owned by white men manage nearly 99% of the $69 trillion U.S. asset management industry, including hedge fund, real estate, private equity, and mutual fund firms (Lerner, 2019). The decisions these white men make about where to invest that money and which companies should survive the crisis will shape the course of society for decades to come. Based on these numbers, it should come as no surprise that companies founded solely by women garnered only 2.2% of the total capital invested in U.S. venture-backed start-ups in 2018—compared with 85% for ventures founded solely by men (Clark, 2018). One industry report found that men comprise nearly 91% of venture-backed start-up founders and 77% of founders are white (RateMyInvestor & DiversityVC, 2019). The high flows of capital into these industries not only raises the top tail of economic inequality, it also limits who makes decisions about which businesses to fund in response to the crisis. Gender, racial, and class disparities in who controls the funding during the coronavirus pandemic will have lasting ramifications for social inequality.

### How Investors Profit on Crisis

Shadow banks have the potential to make investments that address the societal problems sparked by the pandemic. With a culture that celebrates innovation, venture capital is the best equipped to fund the private sector’s pandemic response, including the need for vaccines, contact tracing, widespread testing, protective equipment, home delivery, and remote work. Venture capital investments in health technology have soared, rising by 76% from early 2019 to reach $8.2 billion by the end of March 2020 (Mulready, 2020). Hedge funds are setting records in health care investments, with a focus on biotech firms developing vaccines and treatments for COVID-19 (Winck, 2020). Shadow banks can and do make tremendous investments that can improve societal wellbeing, especially by funding companies that provide essential services such as health care and grocery delivery during the pandemic. But is it effective and fair for these investors to decide what gets funded during a pandemic? And when investors, rather than government stimulus, drive the funding, what are the social and economic costs to society as a whole?

As a start-up traverses the venture capital ecosystem, its potential to innovate and enrich society is often curtailed by investor demands. Venture capital’s fascination with creating “unicorns,” start-ups with valuations over a billion dollars, inspires speculative investments focused on destroying competition and devaluing labor rather than benefiting society (Duhigg, 2020; Kenney & Zysman, 2019). Take the example of Instacart, a grocery delivery platform, which drew early attention from major venture capitalists. As Instacart acquired more investors, including hedge fund and private equity firms, it downgraded the pay structure for its contractors who lack the protections and benefits of full-time employees. Instacart has since earned a reputation as one of the worst employers: Most contractors only earn on average $7.66 per hour (McIntyre, 2019).

As people sheltered-in-place for the pandemic, Instacart became the largest player in the grocery delivery market. The company came under fire for labor concerns as their 200,000 contractors carried out essential work and risked catching COVID-19. In late March, thousands went on strike to demand better safety measures, protective equipment, hazard pay, higher default tips, and expanded sick leave. The “sickout” prompted Instacart to provide hand sanitizer and disinfecting supplies and adjust the tipping system—small efforts that gig labor organizers called “insulting” and “a sick joke” (Dickey, 2020). Then, in early 2021, Instacart laid off more than 1,800 workers, including all of its unionized contractors (O’Brien, 2021). In denying its contractors sufficient protections, benefits, and pay, Instacart prioritized the interests of its investors over those of its employees, putting them at risk during the pandemic.

In addition to maximizing profit by cutting labor costs, shadow banks are profiting on sectors that are flailing because of the crisis. These include industrial and energy products affected by disruptions in global supply chains (Seric et al., 2020). The crisis has decimated the domestic labor, restaurant, and travel and leisure industries, leaving many workers—who are disproportionately Black and Latinx workers—furloughed or out of work entirely. Similarly, workers affected by private investments in education (Gujral et al., 2020) and retirement services (Spanko, 2020) have been hard hit. Shadow banks have advocated for degraded working conditions that made workers in these sectors vulnerable during the crisis (Appelbaum & Batt, 2014; Brav et al., 2013; Strauss, 2014) and also are exploiting these hardships as investment opportunities. Moreover, these struggling sectors, especially the airline, travel, energy, and hospitality sectors, are experiencing significant outflows of capital and selling off of shares. As these companies decrease in value, they become appealing to shadow banks as opportunities for short-selling^2^ (e.g., betting that the stock will decrease in value) and distressed debt investing (e.g., buying cheap stock in companies that have or are likely to file for bankruptcy).

Shadow banks are scouring the economy for flailing companies with depressed stock prices because of the pandemic. Hedge funds aim to profit by buying up low-priced shares and holding the position until the crisis subsides or by shorting the stock. For example, hedge fund investor Carl Icahn has generated a $1.3 billion profit by short-selling stocks in shopping malls hit by COVID-19 restrictions (Cohan, 2020). Meanwhile, the hedge fund Woodson Capital Management more than doubled its portfolio—from $675 million to $1.7 billion—by short-selling brick-and-mortar retailers and investing in e-commerce (Chung, 2020). In other crisis maneuvers, private equity takes public companies private at lower valuations, acquires underperforming corporate subsidiaries to restructure, and buys devalued assets or stocks likely to appreciate after the pandemic.

Hedge funds and other investors are also directing their attention to the potential gains and shortfalls of insurance companies during the pandemic. As insurers pay out claims related to COVID-19, they need more capital on hand (Brush, 2020), which will create a capital shortage and drive up costs in the coming years. Hedge funds are positioning their portfolios accordingly while the insurance assets are low during the crisis. Investors also expect insurance-linked securities to benefit from pandemic premium hikes, with opportunities to profit from catastrophe bonds: high-yield bonds designed to raise money for insurance companies during natural disasters and other crises (Neate & Jolly, 2020).

Meanwhile, other sectors are surging because of pandemic-induced demand. In addition to vaccine and therapy-related stocks, investors are profiting from “work-from-home” stocks—the most notable being Zoom. Companies such as Amazon (online sales) and Staples (home office products) are benefiting from the pandemic, along with utilities, health care, technology and communications. These companies are well-positioned to extract monopoly rents on the crisis, especially Amazon whose founder Jeff Bezos saw his fortune grow by an estimated $71 billion from March to December of 2020 (Collins, 2020). And hedge funds, for one, are profiting from this by upping their holdings in Amazon stock: Amazon became the most widely held stock among hedge funds as the pandemic ensued throughout 2020 (Taub, 2020). Other top hedge fund stocks during the crisis have focused on remote work, e-commerce, packaging, and health care, such as Adobe, Bristol-Myers Squibb, Crown Holdings, Comcast, Salesforce, PayPal, and UnitedHealth Group (Klebnikov, 2020).

Last, shadow banks have a record of profiting on stock market crashes and sociopolitical crises. In 2008, hedge fund managers made billions betting that the housing bubble would burst (Lewis, 2011). In March of 2020, hedge funds capitalized on the stock market crash following the pandemic shutdowns. In mid-February, hedge fund manager Bill Ackman began buying insurance on various bond indexes betting that the debt bubble would burst and U.S. equity and credit markets would subsequently tumble (Cohan, 2020). Ackman took a $27 million position on March 3rd. Then, predicting that the markets would rally alongside a government intervention, he sold his positions on March 23rd, the day the U.S. Federal Reserve announced new measures to support the flailing economy, making a $2.6 billion profit. Ackman was not alone. Miami-based Universa Investments made over a 4,000% return from the early stock market sell-off (Jakab, 2020). It is no coincidence that these mass profits coincided with widespread furloughs and unemployment for average workers.

## Conclusion

The coronavirus pandemic has acted as an incendiary device that has exposed and exacerbated existing economic, racial, gender, and socioeconomic divides in liberal market economies. While investors have provided crucial funding for companies delivering health care innovations, protective equipment, home delivery, and remote work, shadow banks have invested in ways that also exploit the vulnerabilities and inequalities laid bare by the crisis. Moreover, shadow banks have helped to create the conditions that made frontline workers vulnerable in the first place: Their long history of predatory financial behavior has contributed to precarious working conditions and growing economic inequality. The patterns of inequality, and finance’s role in driving them, are not unique to the coronavirus crisis. But the fault lines of the pandemic have revealed how financial capitalism exploits labor and capital through speculative and private channels that are divorced from the real economy.

The coronavirus crisis has revealed what is at stake for society. While the most economically vulnerable have suffered the brunt of the economic and health fallouts from the pandemic, those with financial capital have benefited from investing in beleaguered and essential sectors alike, based on expected opportunistic financial gains arising from the pandemic. This dynamic has created a financialized caste system in which some workers carry out the hard work of fighting the crisis, while another group simultaneously funds and exploits these efforts from the safety of their homes, reaping the rewards because it holds the capital in a rentier capitalist system.

As a result, the economic hardship precipitated by the pandemic has worsened existing gender, racial, and social class inequalities. While men’s employment was more at risk during previous economic upheavals, such as the 2008 financial crisis, the coronavirus pandemic has posed a greater threat to women’s employment (Alon et al., 2020), who have lost the majority of jobs (IWPR, 2020). In December of 2020, the U.S. economy lost 120,000 jobs: Black and Latinx women lost 156,000 jobs while men gained 16,000 (Kurtz, 2021). A recent McKinsey & Company (2020b) survey finds that 57% of Latinx workers and 52% of Black workers, yet only 44% of white workers, report that the pandemic has jeopardized their finances.

While those struggling to make ends meet resort to unemployment and food stamps, U.S. billionaires increased their riches by $1 trillion, over one-third, from March to December of 2020 (Collins, 2020). Even the elite capitalist class will look different after the crisis. The ballooning riches of billionaires such as Jeff Bezos and Elon Musk suggest that the pandemic has boosted the fortunes of billionaires in technology and finance more so than those of industrial billionaires. In light of these stark statistics, a significant question looms: How to control or eradicate the predatory and opportunistic investment behaviors of those with substantial amounts of capital that compromise the wellbeing and livelihoods of the rest of society?

The political and labor domains offer potential solutions that warrant further research. U.S. Senator Elizabeth Warren has proposed sweeping new controls on private investment firms (Stockler, 2019). Calling the negative impact of private equity on wage growth “legalized looting,” Warren has suggested new restrictions on how these firms run companies to curb the adverse consequences for workers. Others have also called for tax and regulatory reform, although enforcement can be challenging (Schneiberg & Bartley, 2010; Soener & Nau, 2019). Some are asking for greater transparency in corporate structures and capital flows, including how companies avoid taxes on investment income (Fligstein & Roehrkasse, 2016). Finally, more attention should be paid to demands for democratization of the workplace (Sobering, 2019) and expanded representation of stakeholders such as workers, consumers, and communities on U.S. corporate boards (Soener & Nau, 2019).

Although the pandemic is a human and economic tragedy of epic proportions, not everyone is suffering to the same extent, and the societal stakes are high. While the economically vulnerable are experiencing vanishing jobs and income, wealthy investors are reaping financial gains from strategic and opportunistic investments. Despite the calls to “build it back better,” the prospects for a more equal and equitable future are bleak. As long as shadow banks can operate in the private sphere with limited oversight and divorced from the real economy, the exploitative and speculative nature of predatory finance ensures that it will continue to capitalize on future crises.

## Notes

1. Whether shadow banks generate a larger return on capital than traditional banks and investment vehicles is a topic of debate: Some research has found that hedge funds, for example, do not outperform the stock market at a sufficient scale to justify the high fees, but insiders often argue that poor performers skew these outcomes (Alpert, 2020).

2. To short a stock, an investor borrows the asset and sells it when the price is high. Then, when the price has dropped, the investor buys the asset, pays the lender back in kind, and pockets the difference in the price.
